# The role of angiogenesis in implant dentistry part II: The effect of bone-grafting and barrier membrane materials on angiogenesis

**DOI:** 10.4317/medoral.21200

**Published:** 2016-03-31

**Authors:** Mohammad-Ali Saghiri, Armen Asatourian, Franklin Garcia-Godoy, Nader Sheibani

**Affiliations:** 1 Bsc, Msc, PhD. Departments of Ophthalmology & Visual Sciences, Biomedical Engineering, and McPherson Eye Research Institute, University of Wisconsin School of Medicine and Public Health, Madison, WI, USA; 2 DDS. Sector of Angiogenesis and Regenerative Surgery, Dr.H Afsar Lajevardi Cluster, Shiraz, Iran; 3 DDS, MS, PhD, PhD. Bioscience Research Center, College of Dentistry, University of Tennessee Health Science Center, TN, USA

## Abstract

**Background:**

In implant dentistry, bone substitute materials and barrier membranes are used in different treatments including guided bone regeneration (GBR), socket preservation, alveolar ridge augmentation, maxillary sinus elevation, and filling bony defects around the inserted dental implant. One of the most important factors in prognosis of treatments using these materials is the growth of new blood vessels in applied areas. Present review was performed to evaluate the effect of the bone-grafting and barrier membrane materials on angiogenesis events.

**Material and Methods:**

An electronic search was performed in PubMed, MEDLINE, and EMBASE databases via OVID using the keywords mentioned in the PubMed and MeSH headings regarding the role of angiogenesis in implant dentistry from January 2000-April 2014.

**Results:**

Of the 5,622 articles identified in our initial search results, only 33 met the inclusion criteria set for this review. Among bone substitute materials the autogenous bone-grafts, and among the barrier membranes the collagenous membranes, had the highest angiogenic potentials. Other bone-grafting materials or membranes were mostly used with pro-angiogenic factors to enhance their angiogenic properties.

**Conclusions:**

Angiogenesis is one of the key factors, which plays a critical role in success rate of GBR technique and is seriously considered in manufacturing bone-grafting and barrier membrane materials. However, there is still lack of clinical and *in-vivo* studies addressing the effect of angiogenesis in treatments using bone-grafting and barrier membrane materials.

**Key words:**Angiogenesis, bone-grafting materials, GBR, ridge augmentation, sinus elevation, socket preservation.

## Introduction

In the field of implant dentistry, bone-grafting and barrier membrane materials can be used in different situation such as the socket preservation (1), alveolar ridge horizontal and/or vertical augmentation, maxillary sinus elevation, and filling the bony defects or exposed threads of implants in order to maintain the required space and provide the necessary time period for migration of regenerative cells to the applied sites (2). The main purpose of using these materials is to prohibit the migration of epithelial or fibroblast cells, and only permit the migration of osteogenic cells into the applied site to regenerate the hard tissue in deficient areas (3,4). The most important principles for increasing the success rate of treatments using these materials is the space maintaining, exclusion of epithelial and connective tissue cells migration to the site, the stabilization of blood clot, and the tight closure of surgical site (5).

Besides these primary surgical principles, blood supply is another crucial factor that provide the required nutritional elements, oxygen, immune system cells, mesenchymal stem cells, and growth factors (6-9). This blood supply is accomplished through angiogenesis, which includes the formation of new blood vessels from preexisting vascular network present in adjacent soft and supraperiosteal tissues (6,10,11). It was shown that the formation of blood vessels through angiogenesis process is an undeniable factor in regenerative procedures such as dentin-pulp complex and dental pulp regeneration (12).

In bone regenerations, angiogenesis plays a central role by providing the functional connection between the grafting-material and surrounding host tissues. The well established and mature vascular networks can assist and accelerate the regenerative processes. In order to promote angiogenesis events, it is suggested to decorticate the surrounding bone to assist with the connection between blood vessels in the bone marrow of adjacent bone and bone substitute materials (6,13).

According to the facts, the present review intended to discuss the angiogenic potential of bone-grafting and barrier membrane materials used in bone regeneration procedures. It was hypothesis that whether bone-grafting and barrier membrane materials promote the angiogenesis event in regeneration and what are the current methods for enhancing the pro-angiogenic effect of these materials.

## Material and Methods

1- The Review Purpose:

Present study was performed to evaluate the effect of different bone-grafting and barrier membrane materials on angiogenesis events during bone regeneration processes in the alveolar bone. The main aspects pursued in this review include: 1) the angiogenic potential of different bone-grafting materials including autogenous, allogenic, xenogeneic, and alloplastic bone materials; 2) the mechanism of action by which these materials can present pro- or anti-angiogenic effects; 3) which one of the bone materials has the highest pro-angiogenic potential; 4) what are the current approaches to enhance the pro-angiogenic effects of these materials; 5) The angiogenic potential of different barrier membranes including collagenous, polymeric, e-PTFE, d-PTFE, titanium-reinforced, and titanium Mesh membranes; 6) which one of these barrier membranes has the highest angiogenic property; and 7) what are the current approaches to enhance the pro-angiogenic effects of resorbable or non-resorbable membranes materials.

2- Inclusion and Exclusion Criteria:

The inclusion criteria were: 1) studies published in English; 2) studies accepted and published between January 2000-April 2014; 3) the scientific *in-vitro, in-vivo,* or *ex-vivo* articles, reviews, systematic reviews, case reports with controlled study design; 4) studies that had evaluated the effect of autogenous, allogenic, xenogeneic, or alloplastic bone-grafting materials on angiogenesis processes in the applied area; 5) studies that had utilized different pro-angiogenic substances in combination with these bone materials; 6) studies that had evaluated the impact of collagenous, polymeric, e-PTFE, d-PTFE, titanium-reinforced, or titanium Mesh membranes on angiogenesis processes in the applied area; and 7) studies that have used different pro-angiogenic agents to enhance angiogenic potential of these membranes. The exclusion criteria were: 1) studies that were published before January 2000 or after April 2014; 2) studies that had not evaluated the angiogenic potentials of the bone-grafting or barrier membrane materials in GBR procedures.

3- Search Methodology.

The searching methodology included electronic searches performed in the PubMed, MEDLINE, and EMBASE databases via OVID using keywords mentioned in the PubMed and MeSH headings including the effects of bone substitute and barrier membranes materials on angiogenesis events occurring in surgical sites after insertion of the dental implant.

4- Search Strategy.

In the electronic search of scientific papers in the PubMed, MEDLINE, and EMBASE databases, the following keywords were used in combination with angiogenesis: “guided bone regeneration”, “autogenous bone graft”, “autogenous bone graft stem cells”, “autogenous bone graft osteoblast”, “autogenous bone graft osteoclast”, “autogenous bone graft endothelial cells”, “allogenic bone graft”, “freeze-dried bone allograft (FDBA)”, “demineralized freeze-dried bone allograft (DFDBA)”, “xenogeneic bone graft”, “alloplastic bone graft”, “resorbable membranes”, “collagenous membranes”, “polymeric membranes”, “non-resorbable membranes”, “e-PTFE membranes”, “d-PTFE membranes”, “titanium-reinforced membranes”, and “titanium mesh membranes”.

## Results

Of the 2,691 articles identified in the initial search results, only 33 met the inclusion criteria set for this review. These 33 studies were directly related to the effect of bone-grafting materials and barrier membrane materials on angiogenesis processes, and are presented in  Table 1 and  Table 1continue . The relevant full text articles and the reference lists of the related articles were evaluated to supplement the search as well. The assessment of the eligibility and finding related data were performed by two reviewers independently. A third reviewer was selected for further discussion and final agreement on any conflict met in the mentioned processes.

**Table 1:**
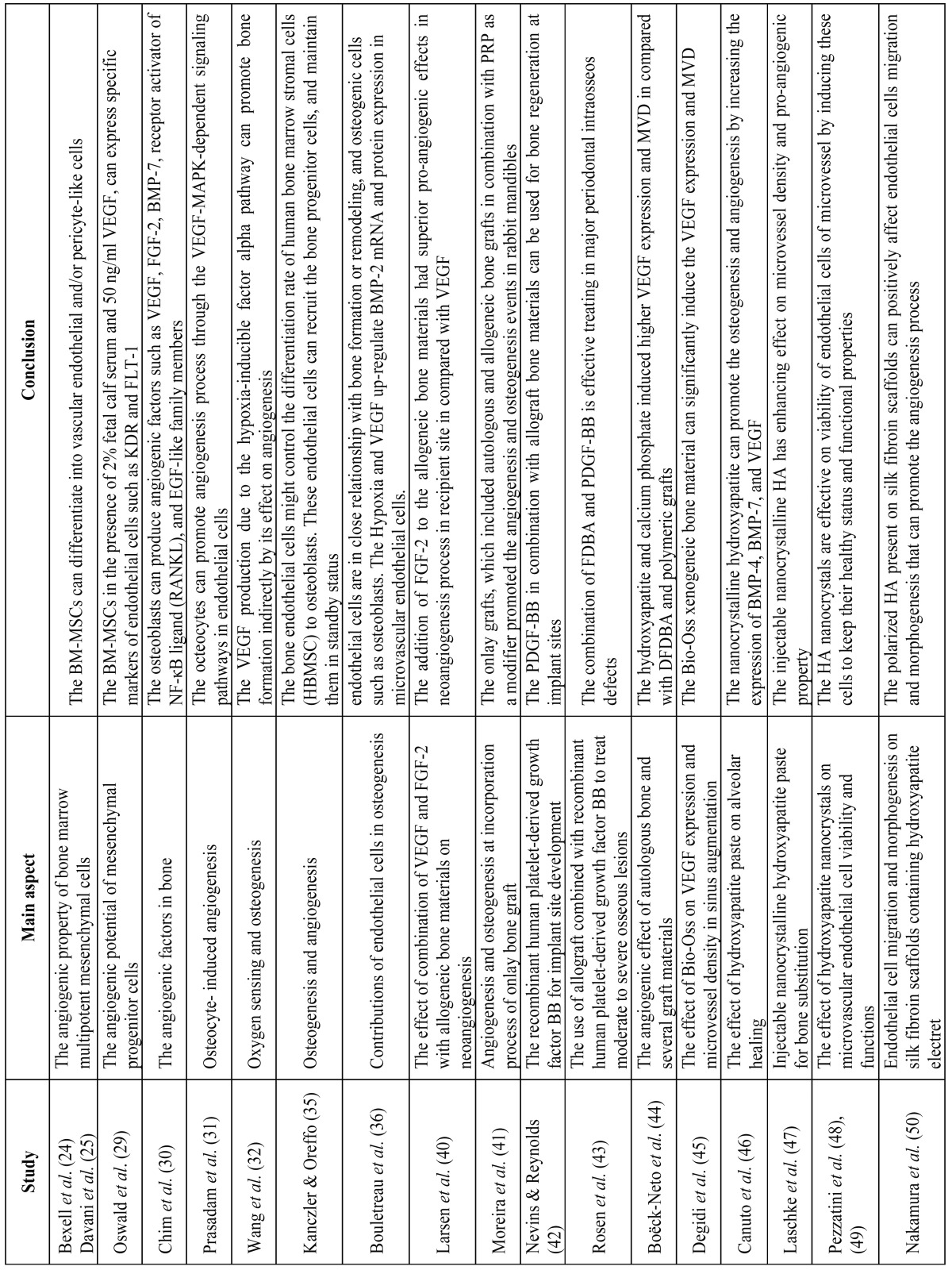
The list of included studies that are directly related to the effect of bone-grafting and barrier membrane materials on angiogenesis process.

**Table 1continue:**
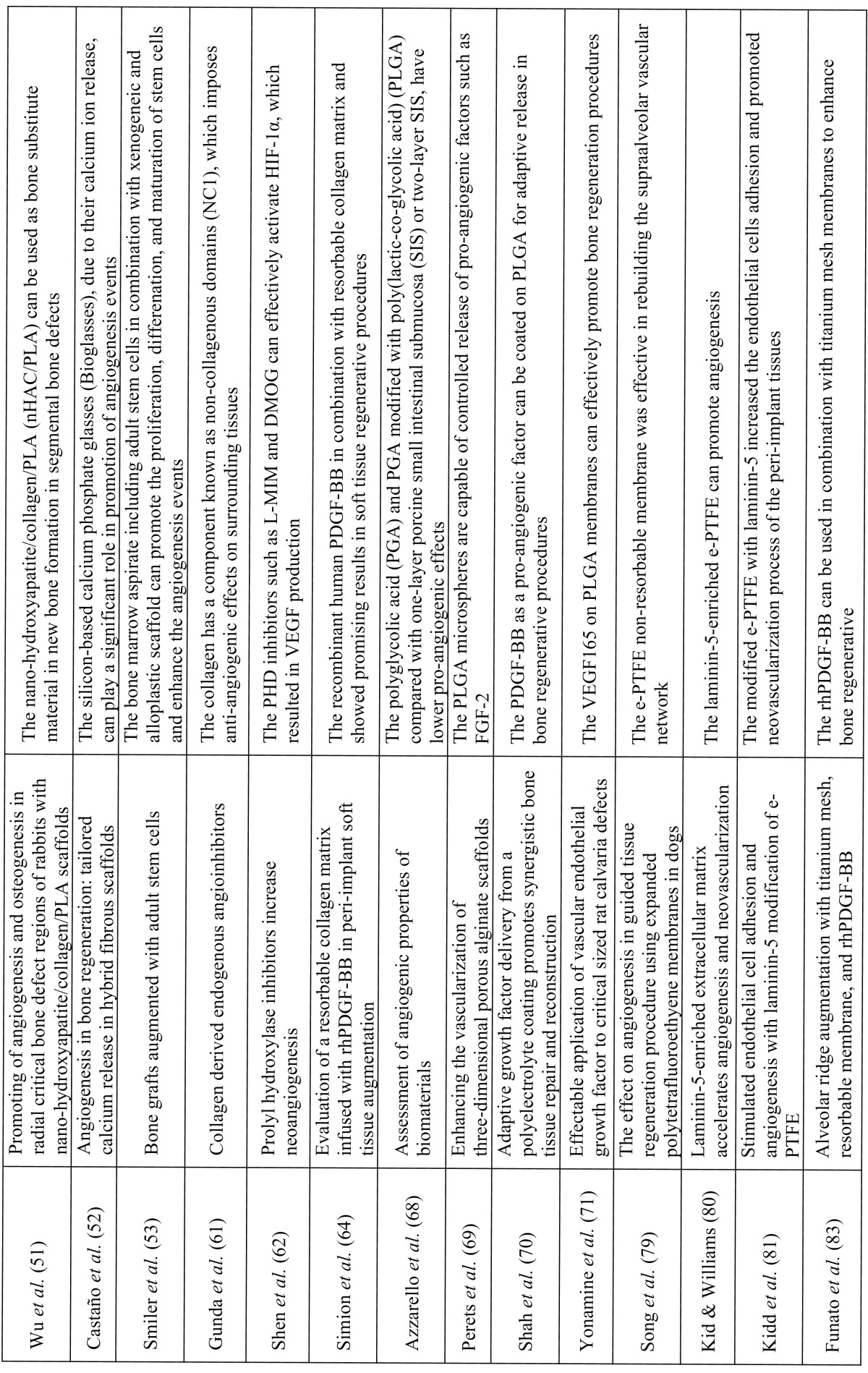
The list of included studies that are directly related to the effect of bone-grafting and barrier membrane materials on angiogenesis process.

## Discussion

1 Effect of bone-grafting materials on angiogenesis

Bone-grafting or bone substitute materials are bio-materials, which are used to replace bone defects (14,15). In 1993 Misch, Dietsh studied different types of bone-grafting materials and based on their mode of action, they categorized them into three different types including autogenous, allograft, and alloplastic materials (16). Three kinds of mode or mechanism of action were introduced as osteogenic, osteoinductive, and osteoconductive. Osteogenic property is the characteristic of a bone-grafting material, which is capable of production and development of new bone even in the absence of undifferentiated mesenchymal stem cells. The osteoinductivity is referred to the materials, which can induce the undifferentiated mesenchymal stem cells present in surrounding bone to differentiate into osteoblast cells and secret and form new bones (17). The last mechanism is the osteoconductivity, which is related to the materials that only provide an inert scaffold or matrix for growth and development of surrounding undifferentiated mesenchymal stem cells (6). Basically, autogenous grafting materials are osteogenic, osteoinductive, and osteoconductive. While allografts have osteoinductivity and osteoconductivity, and the alloplasts and xenografts only have osteoconductivity (6,16). The application of these grafting materials mainly depends on the size of bone defect and the topography of applied area (16). In the following sections the angiogenic effects of autogenous, allograft, alloplast, and xenograft materials are overviewed.

2.1- Autogenous bone materials 

These bone-grafting materials are provided in block or particulate forms from other locations in the body of the same individual who is subjected to dental implant surgery. These donor sites can be intraoral such as ramus, mandibular symphysis, maxillary tuberosity, and tori or exostoses. The extraoral areas are mainly the tibial plateau or iliac crest (6). Autogenous materials mostly used for larger bony defects, where three or more walls are lost (16). These materials are osteogenic, osteoinductive, and osteoconductive, which are considered as material of choice for large bony reconstructions (16).

The most important point about the autogenous bone materials is the similarity of these grafting materials to the applied bony area. In other word, whatever the defected bone contains, the same substances can be found in the autogenous grafting material. These grafts have three components including: 1) a mature bony structure that serves as a physical matrix; 2) viable cells such as osteocytes, osteoclasts, bone marrow-derived mesenchymal stem cells (BM-MSCs), and also endothelial cells that might be neglected among these cells; and 3) growth factors and cytokines necessary for hard tissue regeneration (6).

The bony structure of autografts can be either cortical, trabecular (cancellous), or corticotrabecular bone. Cortical type grafts have more condensed structure with higher levels of growth factors such as bone morphogenic proteins (BMP), while the trabecular types have more viable cells (18). The physical matrix of autogenous grafts has lesser effects on angiogenesis processes compared to the cellular and growth factor components. Regarding this issue, authors indicated that cortical bone grafts compared to the cancellous type grafts have more resistance against the penetration of the newly formed vessels from the recipient bed into the graft material (19-21).

The second component of autogenous grafts is the viable cells inside the graft material. Among these cells, the most important ones are the BM-MSCs, which provide osteogenicity through differentiation into bone progenitor cells (22). These cells are capable of self-renewal and differentiation into osteogenic cells (23). The angiogenic effects of these cells include the differentiation into vascular endothelial and/or pericyte-like cells (24,25). The other pro-angiogenic effect is through secretion of vascular endothelial growth factor (VEGF) or fibroblast growth factor-2 (FGF-2) (26-28). Oswald *et al.* reported that BM-MSCs, in the presence of 2% fetal calf serum and 50 ng/ml VEGF, can express specific markers of endothelial cells such as KDR and FLT-1 (29) ( Table 1 and  Table 1continue).

The other resident cells are the osteoblasts, osteocytes, and osteoclasts which have remarkable role in angiogenesis events. Chim *et al.* showed that these cells can produce angiogenic factors such as VEGF, FGF-2, BMP-7, receptor activator of NF-κB ligand (RANKL), and epidermal growth factor (EGF)-like family members (30). Prasadam *et al.* indicated that octeocytes can promote angiogenesis through the VEGF-MAPK-dependent signaling pathways in endothelial cells (31). Wang *et al.* demonstrated that the VEGF production due to the hypoxia-inducible factor alpha (HIF-1α) pathway can promote bone formation indirectly by its effect on angiogenesis (32) ( Table 1 and  Table 1continue).

Endothelial cells are the other viable cells present in autogenous grafts. Authors indicated that bone endothelial cells have specific characteristics such as responding to estrogen, PTH, and cytokines (33,34). It was reported that endothelial cells might control the differentiation rate of human bone marrow stromal cells (HBMSC) to osteoblasts (35). Investigators showed that endothelial cells can recruit the bone progenitor cells, and maintain them in a standby status before their migration to required site, and promote their differentiation into functional osteoblasts after migration from blood vessels (35). The hypoxia induced pro-angiogenic factors such as VEGF can promote the expression of BMP-2 in endothelial cells. These results indicate that endothelial cells are in close relationship with bone formation or remodeling, and osteogenic cells such as osteoblasts (36) ( Table 1 and  Table 1continue,  Table 2).

**Table 2:**
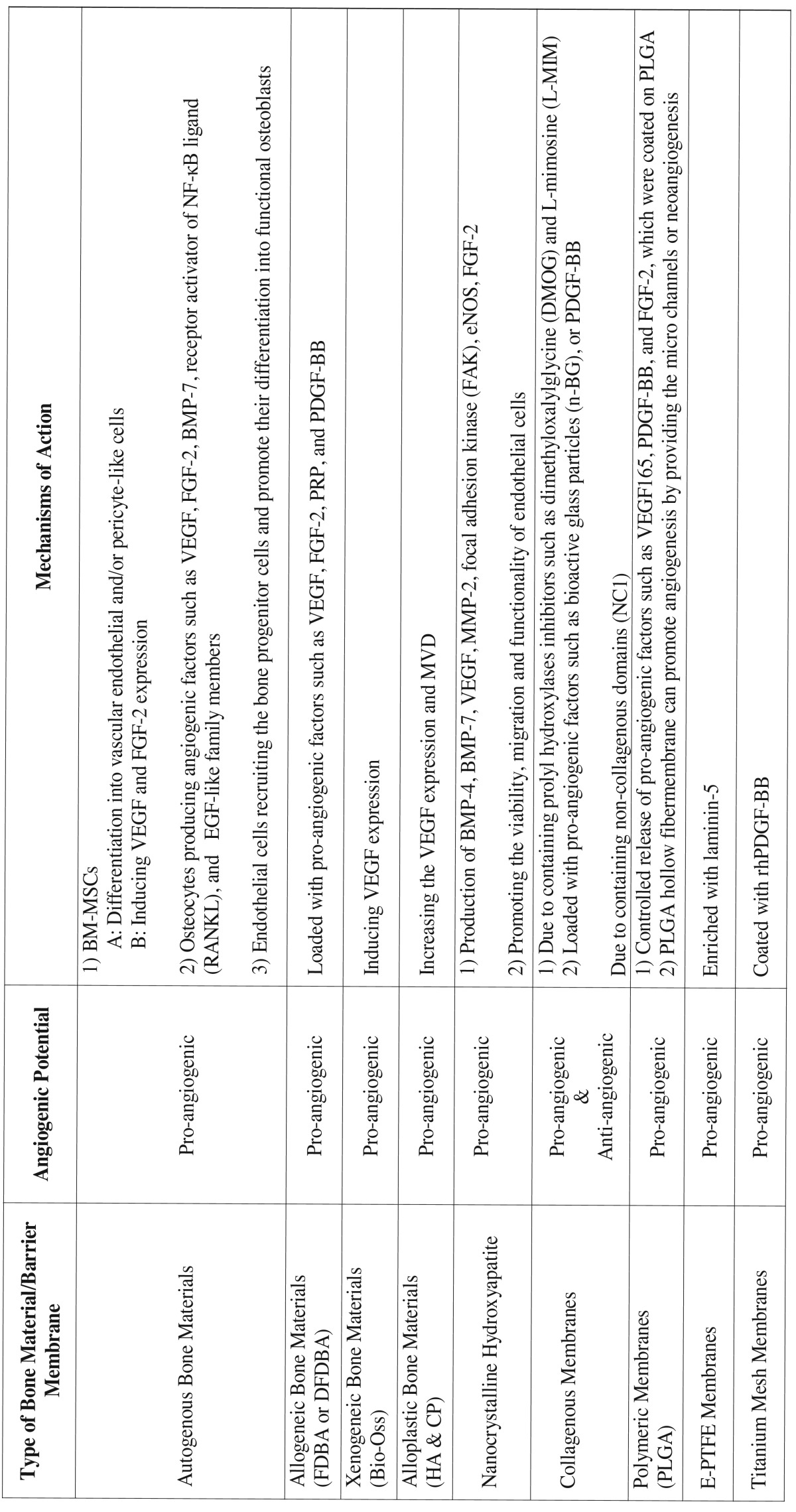
The angiogenic potentials of bone and barrier membrane materials used in GBR procedures.

2.2- Allogenic bone materials

Generally the allogenic grafting materials are tissues derived from one individual and used as graft in another individual with different genetic traits. These materials are mostly considered to be osetoinductive and osteoconductive and do not express any osteogenic characteristic, as autogenous bone grafts do (6). In order to eliminate the risk of cross contamination, two methods of freeze-drying and the Tutoplast® processing are utilized to prepare allogenic bone grafts (37,38). The materials provided by freeze-drying technique include the demineralized freeze-dried bone allograft (DFDBA), which is osteoinductive and osteoconductive with faster resorption rate; and the freeze-dried bone allograft (FDBA) that is osteoconductive with slower resorption rate (39).

**Figure 1:**
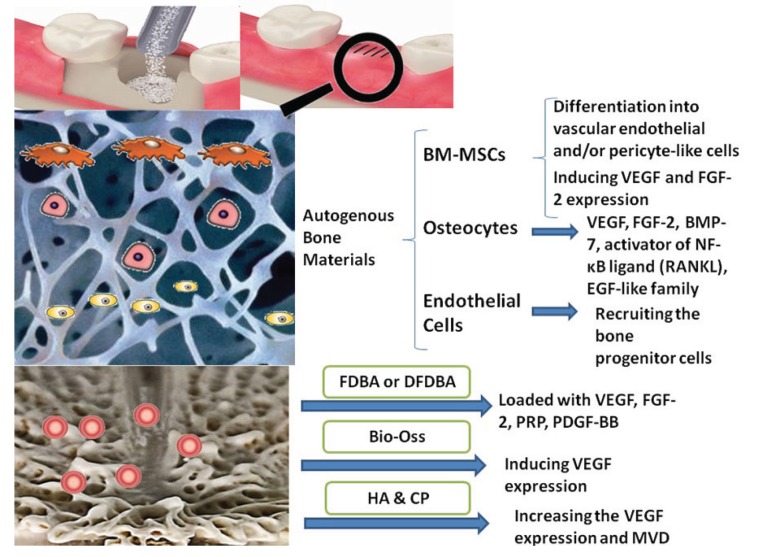
The angiogenic effects of bone-grafting materials in bone regenerative procedure in schematic socket preservation illustration. Upper figure: the procedure of socket preservation using bone substitute material; Middle figure: presents a schematic figure of autogenous bone material including valuable cellular components such as BM-MSCs, osteocytes, and endothelial cells; Bottom figure: presents a schematic figure of allograft, xenograft, or allopalastic materials, which only includes a matrix and incorporated growth factors (pink spots).

Larsen *et al.* reported that the neoangiogenesis at a recipient site is critical for survival of vascularized allogenic bone grafts (Fig. 1). These authors evaluated the effects of VEGF, FGF-2, and the combination of these pro-angiogenic factors as an additive to allogenic bone materials, on the angiogenesis events occurring at grafting site (40). They showed that FGF-2 had superior pro-angiogenic effects in neoangiogenesis process at the recipient site compared with VEGF (40). Moreira *et al.* indicated that onlay grafts, which included autogenous and allogenic bone grafts in combination with platelet-rich plasma (PRP) as a modifier, promoted the angiogenesis and osteogenesis events in rabbit mandibles (41) ( Table 1 and  Table 1continue).

Angiogenesis plays a significant role in osteoblast differentiation and bone matrix formation at grafted sites (41). Nevins, Reynolds showed that platelet derived growth factor-BB (PDGF-BB) in combination with allograft bone materials can be used for bone regeneration at implant sites (42). It was indicated that the PDGF-BB, which is a pro-angiogenic factor, with FDBA or DFDBA as a scaffold, can be utilized for alveolar bone augmentation (42). Similar results were reported by Rosen *et al. * that showed in a retrospective case series report, of the effective combination of FDBA and PDGF-BB in major periodontal intraosseos defects (43). Boëck-Neto *et al.* evaluated the VEGF expression and microvessel density (MVD) after maxillary sinus augmentation with different allograft, alloplastic grafting materials (44). It was indicated that DFDBA and polymeric grafts induced the lowest level of VEGF expression and MVD, while the hydroxyapatite (HA) and calcium phosphate (CP) showed the highest values (44) ( Table 1 and  Table 1continue,  Table 2).

The combination of other pro-angiogenic factors such as BMPs, angiopoietins, matrix metalloproteinase (MMP), or stem cell factors (SCF) with allogenic grafts can be taken into consideration in future studies.

2.3- Xenogeneic and alloplastic bone materials 

Xenogeneic bone substitute materials are grafting materials obtained from other non-human species such as bovine. These substances are natural HA or anorganic bone matrix (ABM), which are obtained from the natural bone of bovine or other animal sources (6). The other grafting materials are the alloplastic substances, which are synthetic graft materials and include bioactive glass polymers, calcium carbonate, calcium sulfate, and synthetic ceramic materials like tricalcium phosphate (TCP) and HA (6). Both xenogeneic and alloplastic materials are considered as osteoconductive substances (Fig. 1), which only provide a physical matrix for recipient cells to infiltrate into the graft and form the new hard tissue (16).

Degidi *et al.* showed that a xenogeneic bone substitute material could significantly increase the MVD after six months. This grafting material could also induce VEGF expression when is used for sinus augmentation (45) ( Table 1 and  Table 1continue,  Table 2).

As previously mentioned, Boëck-Neto *et al.* showed that the alloplastic ceramic materials like HA and calcium phosphate induce the highest amount of VEGF expression and (MVD) in maxillary sinus lifting surgery compared with DFDBA and other alloplastic materials such as polymers (44). Canuto *et al.* indicated that nanocrystalline hydroxyapatite can promote the osteogenesis and angiogenesis by increasing the expression of BMP-4, BMP-7, and VEGF (46). Laschke *et al.* suggested that injectable nanocrystalline HA bone grafting materials such as Ostim due to their enhancing effect on microvessel density and pro-angiogenic property, can be used for guided vascularization procedures (47) ( Table 1 and  Table 1continue,  Table 2). Pezzatini *et al.* demonstrated that HA nanocrystals can enhance angiogenesis by inducing the migration of endothelial cells and increasing the secretion of matrix metalloproteinase-2 (MMP-2), activation of focal adhesion kinase (FAK), endothelial nitric oxide synthase (eNOS), and FGF-2 expression (48). In another study, Pezzatini *et al.* indicated that HA nanocrystals are effective on viability of endothelial cells of microvessel by inducing these cells to keep their healthy status and functional properties (49). Nakamura *et al.* reported that polarized HA present on silk fibroin scaffolds can positively affect endothelial cells migration and morphogenesis, which results in promotion of angiogenesis events (50). Wu *et al.* demonstrated that nano-hydroxyapatite/collagen/PLA (nHAC/PLA) can be used as bone substitute material in new bone formation in segmental bone defects, due to its enhancing effect on angiogenesis and osteogenesis processes (51).

Castaño *et al.* indicated that silicon-based calcium phosphate glasses (Bioglasses), due to their calcium ion release, can play a significant role in promotion of angiogenesis events (52).

Smiler *et al.* used bone marrow aspirate including adult stem cells in combination with xenogeneic and alloplastic scaffold in bone regeneration. These authors showed that these combinations can promote the proliferation, differenation, and maturation of stem cells and enhance the angiogenesis events (53).

2.4- Effect of barrier membranes on angiogenesis 

In 1988, Dahlin *et al.* proposed the GBR procedure protocol, which included the surgical placement of a barrier membrane on the subjected bony area to seal and provide the required space for bone regeneration (54). This study apparently showed the importance of using barrier membranes and their functional role in bone regenerative procedures. The most important purpose for using barrier membranes is to create a space on defected bone in order to only permit the bone progenitor cells to migrate into this space, and prevent the in-growth of soft tissue cells into the defective area (54).

Barrier membranes are basically divided into two categories of resorbable and non-resorbable membranes. In the following sections the anigogenic properties of these membranes are overviewed.

2.5- Resorbable membranes

Resorbable membranes are barrier membranes, which after a short time are resorbed by hydrolysis or catabolic reactions, and do not need to be removed from the grafted site (55). However, these membranes, due to the fast biodegradation, might not be useful for regeneration procedures that require the physical space maintenance for more than one month (56). Resorbable, membranes are categorized under two main groups of collagenous and polymeric membranes.

2.6- Collagenous membranes

Collagenous membranes are provided from type I or combination of type I and III collagens, which is obtained from pericardium, skin, or tendons of human, procine, or bovine (57). Collagen membranes are considered as one of the ideal membranes for regenerative procedures due to their superior biocompatibility and bioactivities such as direct effect on bone formation (58), and chemotactic effects on periodontal ligament (PDL) or gingival fibroblasts (59,60).

Gunda *et al.* indicated that collagen has a component known as non-collagenous domains (NC1), which imposes anti-angiogenic effects on surrounding tissues (61). Shen *et al.* reported that prolyl hydroxylase enzyme (PHD) is a key enzyme with central role in degradation of hypoxia inducible factor alpha (HIF-1α), which is an angiogenic initiating factor in tissue development, regenerations, and reparations (62). These authors showed that PHD inhibitors such as L-MIM and DMOG can effectively activate HIF-1α, which resulted in VEGF production (62).

Vargas *et al.* indicated that addition of 10 wt% nano-sized (20-30 nm) bioactive glass particles (n-BG) to the bovine type I collagen can promote the angiogenic characteristics of collagenous composites, which are used in tissue engineering procedures (63). Simion *et al.* used the pro-angiogenic factor, recombinant human PDGF-BB, on resorbable collagen matrix and reported promising results in soft tissue regenerative procedures (64) ( Table 1 and  Table 1continue,  Table 2).

2.7- Polymeric membranes

Polymeric membranes are the second category of resorbable membranes, which are synthetic membranes composed of polyglycolides (PGAs), polylactides (PLAs), polyesters, and co-polymers (65). One of the disadvantages of polymeric membranes compared to collagenous membranes is the provocation of host inflammatory responses, which is much higher in case of polymeric membranes (66). However, polymeric membranes are mostly degraded by hydrolysis reaction that reduces the pH value and produces an acidic condition, which negatively impacts bone regeneration process (67).

Azzarello *et al.* indicated that polyglycolic acid (PGA) and PGA modified with poly(lactic-co-glycolic acid) (PLGA) compared with one-layer porcine small intestinal submucosa (SIS) or two-layer SIS, have lower pro-angiogenic effects (68). Perets *et al.* suggested that PLGA microspheres are capable of controlled release of pro-angiogenic factors such as FGF-2. By incorporating this angiogenic factor, the PLGA membrane promoted the angiogenesis processes at grafted areas (69). Shah *et al.* showed that PDGF-BB as a pro-angiogenic factor can be coated on PLGA for adaptive release in bone regenerative procedures (70). Yonamine *et al.* reported that the incorporation of VEGF165 on PLGA membranes can effectively promote bone regeneration procedures (71). Ellis, Chaudhuri demonstrated that the PLGA hollow fibermembrane can promote angiogenesis by providing the micro channels inside its structure that permit angiogenesis to occur in these channels (72) ( Table 1 and  Table 1continue,  Table 2).

2.8- Non-resorbable membranes:

Non-resorbable membranes are other types of membranes, which require the clinician to remove them after application at grafted areas. These membranes are mostly contaminated with bacteria and must be removed within 4-6 weeks after surgical application. Non-resorbable membranes include different types such as expanded polytetrafluoroethylene (e-PTFE), high-density polytetrafluoroethylene (d-PTFE), titanium-reinforced PTFE, and titanium mesh membranes (73). In the following section the anigogenic properties of these membranes are discussed.

2.9- E-PTFE, d-PTFE, and titanium-reinforced PTFE membranes

E-PTFE membranes have become a standard membrane for GBR procedures since 1990s (74-76). Becker *et al.* indicated that e-PTFE membranes can be successfully used for bone regeneration procedures around dental implants (76). The other type of PTFE membranes, is the d-PTFE membrane, which has higher density and smaller pore size (> 0.3 µm) compared to e-PTFE membranes with larger pore sized (5-20 µm) (77,78). D-PTFE membranes beside their higher density, have another advantage over e-PTFE membranes, which is their use in situations that the primary soft closure is not possible. In bone regeneration procedure such as socket preservation or other conditions, which due to tissue tension the primary closure is not affordable, the d-PTFE membranes can be used safely (78). Titanium-reinforced PTFE membranes contain a titanium framework embedded in e-PTFE or d-PTFE membranes, which enable them to be shaped easily and maintain their shape at surgical site. These membranes can be used in larger bony defects without rebounding or collapsing into the defective areas (6).

Song *et al.* used the e-PTFE membrane for regeneration of mandibular boney defects and evaluated its effects on angiogenesis processes at 2, 3, 4, and 8 weeks. These authors noticed that the presence of e-PTFE non-resorbable membrane was effective in rebuilding the supraalveolar vascular network. However, it did not affect vascular anastomosing between new connective tissue and gingival flap, while 2-4 weeks after removal of membranes the vascularization between the gingival flap and new connective tissue became normal (79) ( Table 1 and  Table 1continue).

Kid, Williams showed that laminin-5-enriched extracellular matrix can promote angiogenesis. These authors used e-PTFE samples, which were modified with cell matrices that were enriched with laminin-5. They noticed that the density of blood vessels was increased significantly (80). In another study Kid *et al.* indicated that the modified e-PTFE with laminin-5 increased the endothelial cells adhesion and promoted neovascularization process of the peri-implant tissues (81) ( Table 1 and  Table 1continue).

2.10- Titanium Mesh membranes

Titanium mesh membranes are another type of non-resorbable membranes, which have a great ability to maintain the space required for alveolar bone augmentation. These membranes can perfectly withstand the pressure of overlying soft tissue and keep a large space for bone regeneration without collapsing (82). Funato *et al.* reported that rhPDGF-BB can be used in combination with titanium mesh membranes to enhance bone regenerative procedures in vertical ridge augmentation (83) ( Table 1 and  Table 1continue).

## Conclusions

In this review the effects of bone-grafting and barrier membrane materials on the angiogenesis processes at recipient sites were overviewed. According to the reviewed studies the following conclusions were drawn.

Autogenous grafting materials have the highest potential for inducing angiogenesis events at the recipient site. The angiogenic properties of these materials are closely related to the viable cells such as BM-MSCs, osteocytes, and endothelial cells.

The angiogenic properties of allogenic bone materials are lower than other grafting bone substitutes. The addition of different pro-angiogenic factors such as VEGF, FGF-2, and PDGF can be promising in increasing the angiogenic activity of these materials.

Among the xenogeneic and alloplastic bone materials, the HA and calcium phosphate have the highest pro-angiogenic effects. The modifications such as nano-sized HA crystals or the combination of calcium phosphate and Bioactive glass might enhance the pro-angiogenic activity in grafted areas.

Resorbable collagenous membranes had pro-angiogenic effects due to release of prolyl hydroxylase inhibitors (L-MIM and DMGO), while the NC1 component in these membranes act as an anti-angiogenic agent. The recent trend includes the enrichment of collagenous membranes and scaffolds with PDGF-BB or nano-sized bioactive glass to enhance their angiogenic properties.

The polymeric membranes do not have any remarkable inherent pro-angiogenic effects and are mostly used as scaffolds for delivering and controlled release of pro-angiogenic factors.

The angiogenic properties of e-PTFE, d-PTFE, titanium-reinforced PTFE, and titanium mesh membranes have not been well-discussed in the literature. However, these non-resorbable membranes might have some angiogenic potential, but most of the pro-angiogenic effects of these membranes are related to the incorporated pro-angiogenic agents, which are used to enhance the angiogenic activity
